# A prediction model for the risk of developing mild cognitive impairment in older adults with sarcopenia: evidence from the CHARLS

**DOI:** 10.1007/s40520-025-02980-2

**Published:** 2025-03-08

**Authors:** Xinyue Liu, Jingyi Ni, Baicheng Wang, Rui Yin, Jinlin Tang, Qi Chu, Lianghui You, Zhenggang Wu, Yan Cao, Chenbo Ji

**Affiliations:** 1 Nanjing Women and Children’s Healthcare Institute, Women’s Hospital of Nanjing Medical University, Nanjing Women and Children’s Healthcare Hospital, Nanjing, Jiangsu China; 2 School of Nursing, Nanjing Medical University, Nanjing, Jiangsu China; 3 Sunshine Union Hospital, Weifang, Shandong China

**Keywords:** Mild cognitive impairment, Sarcopenia, CHARLS, Prediction model, Morbidity probability, Online tool

## Abstract

**Background:**

Sarcopenia significantly increases the risk of cognitive impairments in older adults. Early detection of mild cognitive impairment (MCI) in individuals with sarcopenia is essential for timely intervention.

**Aims:**

To develop an accurate prediction model for screening MCI in individuals with sarcopenia.

**Methods:**

We employed machine learning and deep learning techniques to analyze data from 570 patients with sarcopenia from the China Health and Retirement Longitudinal Study (CHARLS). Our model forecasts MCI incidence over the next four years, categorizing patients into low and high-risk groups based on their risk levels.

**Results:**

The model was constructed using CHARLS data from 2011 to 2015, incorporating eight validated variables. It outperformed logistic regression, achieving an Area Under the Curve (AUC) of 0.708 (95% CI: 0.544–0.844) for the test set and accurately classifying patients’ risk in the training set. The deep learning model demonstrated a low false-positive rate of 10.23% for MCI in higher-risk groups. Independent validation using 2015–2018 CHARLS data confirmed the model’s efficacy, with an AUC of 0.711 (0.95 CI, 0.588–0.823). An online tool to implement the model is available at http://47.115.214.16:8000/.

**Conclusions:**

This deep learning model effectively predicts MCI risk in individuals with sarcopenia, facilitating early interventions. Its accuracy aids in identifying high-risk individuals, potentially enhancing patient care.

**Supplementary Information:**

The online version contains supplementary material available at 10.1007/s40520-025-02980-2.

## Introduction

The progression of cognitive function in humans typically follows a trajectory from normal age-related cognitive decline to mild cognitive impairment (MCI), and eventually to Alzheimer’s disease [1]. MCI is characterized by both subjective and objective decline in one or more cognitive domains, such as language, memory, calculation, and orientation, compared with baseline function, without disability in instrumental activities of daily living (IADL) [2]. However, MCI is marked by an insidious and often undetectable onset, rapid progression, easy evolution to dementia, and the absence of effective pharmacological treatment. According to the clinical practice guideline on MCI issued by the American Academy of Neurology, 14.4-55.6% of individuals with MCI may regain neurological integrity [3], thereby reducing the occurrence of dementia. Consequently, the accurate and rapid identification of significant cognitive decline and the onset of MCI is crucial for the prevention of dementia. However, the factors contributing to cognitive decline are numerous and complex, and we are unable to accurately predict the extent of cognitive decline, the risk of developing MCI, and etiologic prevention for those at high risk in all populations.

In patients with sarcopenia, the prevalence of MCI is 24.2% [4]. Studies have shown that individuals with sarcopenia is 1.72 times more likely to develop MCI than those without sarcopenia, and that patients with MCI experience an annual dementia progression rate of 10–20% [5]. This study focuses on the sarcopenia population, which has a high prevalence of MCI and is associated with severe adverse outcomes. Defined as a progressive and widespread accelerated loss of muscle mass and muscle function [4], sarcopenia is now formally recognized as a muscle disease [6], with an increasing prevalence in the elderly population every year. It is considered an independent risk factor for MCI.

There are shared physiological, and pathological mechanisms between sarcopenia and MCI. Common metabolic factors (chronic inflammation, oxidative stress [7]), behavioral factors (exercise [8], socialization), and psychological factors (depressive symptoms [9]) can affect both the body and cognition. The association between the two conditions has been well studied. First, evidence suggests that age-related loss of muscle mass, function, and strength occurs before cognitive decline. Thus, sarcopenia and its components serve as predictors of future cognitive impairment [10]. Second, numerous prior longitudinal studies have also shown that the components of sarcopenia, primarily low grip strength [11, 12] and slow gait speed [13], are significantly associated with the onset of MCI and can predict cognitive decline. Finally, the cognitive decline associated with sarcopenia is thought to somewhat independent of the nervous system, muscles may influence cognition through hormone-like proteins secreted by the muscles [14]. Therefore, while direct mechanistic studies linking sarcopenia and MCI are still lacking, it is feasible to predict the risk of MCI using simple physical measures, which can assist clinicians in decision-making and guide interventional treatments.

We have not yet retrieved the prediction of MCI based on sarcopenia-related characteristics and clinical metrics, and other factors. To address this issue, we developed a risk prediction model for MCI in sarcopenia using the CHARLS cohort. The increased computational power and the availability of big data have facilitated the application of deep learning (DL) to healthcare [15], distinguishing it from simple regression by enabling the analysis of nonlinear relationships between characteristic variables and outcomes. In this study, we employed a DL model to predict risk of MCI in patients with sarcopenia.

## Methods

The China Health and Retirement Longitudinal Study (CHARLS) is organized by the China Social Science Survey Center at Peking University. Its goal is to collect a set of high-quality microdata representative of China’s middle-aged and elderly households and individuals aged 45 years and older and to promote interdisciplinary research on the issue of aging. The CHARLS national baseline survey was carried out in 2011 and is tracked every two to three years. The study covering 150 counties, 450 villages, and approximately 17,000 individuals across 10,000 households. To date, CHARLS has released five national surveys.

### Participants

**Fig. 1:**
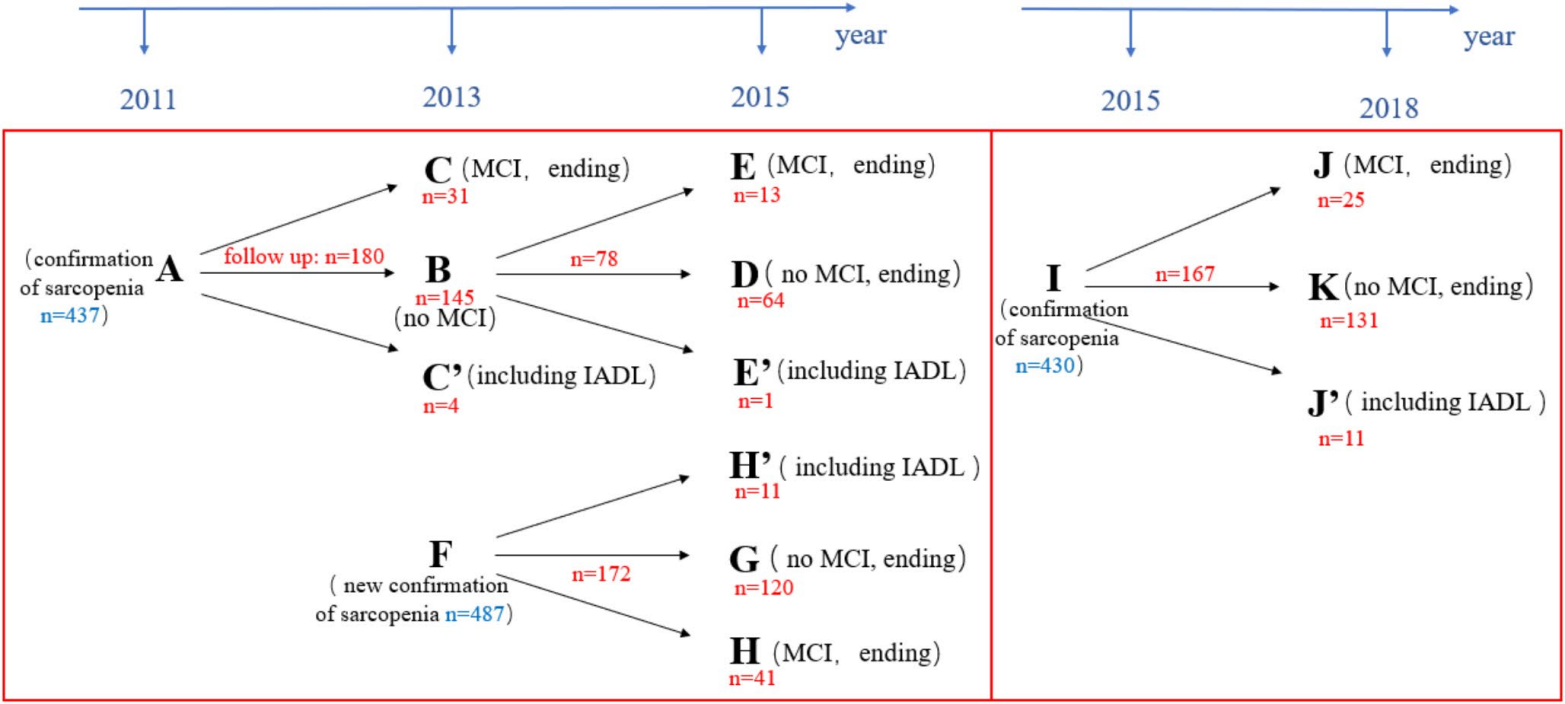
Sample screening flowchart

We used data from four prior national surveys (2011, 2013, 2015, 2018) to collect physical measures of patients with sarcopenia. Only longitudinal data with normal cognitive function at baseline measurement and with indicators of cognitive and activities of daily living (ADL) outcomes were included. We excluded individuals with severe physical impairments and psychiatric disorders. A total of 570 patients with sarcopenia were included in our subsequent analysis. Detailed information about CHARLS is available in previous literature [16]. All CHARLS datasets can be downloaded at the CHARLS home page at http://charls.pku.edu.cn/en. The CHARLS survey project was approved by the Biomedical Ethics Committee of Peking University, and all participants were required to sign informed consent. Figure 1 presents the sample screening flowchart. Additional Details on data screening can be found in the supplementary material.

### Assessment of sarcopenia

According to the Asian Working Group for Sarcopenia (AWGS 2019), sarcopenia is defined as loss of muscle mass, as well as low muscle strength and (or) poor physical performance [17]. We use Appendicular Skeletal Muscle (ASM) to represent muscle mass. ASM is generally measured using the Dual-energy X-ray Absorptiometry (DXA) or Bioelectrical Impedance Analysis (BIA). Chinese ASM can be evaluated using this formula: ASM = 0.193 × weight (kg) + 0.107 × height (cm) − 4.157 × gender − 0.037 × age (years) − 2.631, which is set to 1 if gender is male and 2 for female [18]. It was shown that the ASM calculated using this formula agrees well with DXA [19]. Low muscle mass was judged based on the 20% of the study population with the lowest gender specificity for height-adjusted muscle mass (SMI = ASM/height2) [4, 20]. Muscle strength was assessed by averaging two maximal grip strengths in both hands. The AWGS defined low muscle power as < 28 kg for men and < 18 kg for women [17]. Physical performance was assessed by walking speed, five-chair stand tests, and the Short Physical Performance Battery (SPPB), where SPPB consists of three tests in standing balance in addition to the first two tests, with 4 points each for a total of 12 points [21]. According to the AWGS 2019 recommendations, low physical performance was defined as gait speed < 1.0 m/s, five-chair stand tests ≥ 12s, or an SPPB score < 9 [17].

### Assessment of MCI

Cognitive function was measured according to the methodology used in the American Health and Retirement Study (HRS) [22]. Participants underwent a face-to-face assessment of four dimensions of cognitive functioning, namely orientation (5 points), memory (10 points), numeracy (5 points), and drawing ability (1 point) [23], for a total score of 21 points. Aging-Associated Cognitive Decline (AACD) was used to define cognitive decline as being at least 1 standard deviation (SD) below the age norm [24]. Participants were grouped every 5 years of age, and participants in each age group who met the AACD criteria were categorized as having a decline in one or more cognitive dimensions [4].

IADL was defined as dependence on or the need for assistance in at least one of the following tasks: housework, cooking, shopping, managing money, and taking medication [25]. For each IADL item, participants selected one of the following four responses: (1) No, I do not have any difficulty; (2) I have difficulty, but I can still do it; (3) Yes, I have difficulty and need help; (4) I cannot do it. A score of 3 or 4 on any IADL item was considered indicative of disability [26].

Participants who met both AACD criteria and had no disability in IADL were classified as having MCI.

### Covariates

We also considered demographic factors, health-related factors and follow-up time that influenced MCI. Demographic characteristics included gender, age, place of residence (urban/rural), education level (primary and below primary school/middle school/high school or vocational school/college degree or higher.), and marital status (partnered/unpartnered) [27]. Health-related factors included history of smoking (yes/no), history of alcohol consumption (yes/no), daily sleep duration (in hours), depressive symptoms (yes/no), history of falls (yes/no), body mass index (BMI), and twelve chronic diseases associated with MCI [28] (hypertension, dyslipidemia, hyperglycemia, chronic lung disease, heart disease, liver disease, kidney disease, digestive system disease, malignant tumor, stroke, arthritis rheumatism, asthma). Among them, BMI was defined as weight (in kg) divided by the square of height (in m). Depression was assessed using the 10-item Center for Epidemiologic Studies Depression Scale (CESD-10), with a total score of 30, with a score of more than 10 suggesting the presence of depressive symptoms [16, 29].

### Statistical analysis

Continuous variables were expressed as means ± standard deviations. Categorical variables were expressed as frequency (n) and proportion (%). A T-test was used to compare the measurement data between the two groups. Multi-group categorical data were compared using the rank sum test. We use Stata software to merge and filter the data. Data were filled with the R software missforest package. A feed-forward neural network model was constructed using Pytorch. The Receiver Operating Characteristic (ROC) curve represented the model’s discrimination and was assessed by the AUC value. A two-sided P value less than or equal to 0.05 was considered statistically significant. Statistical analyses were performed using Stata17, R.4.2.2, and Pytorch 2.2.1.

## Results

### Demographic characteristics of participants

570 patients with sarcopenia met the inclusion criteria, of which 110 had MCI. Table 1 provides the demographic characteristics of the participants. The average age of the sample population was 69.22.

**Table 1:** Demographic characteristics of the participants

Variables	Total(*n* = 570)
Age	69.22 ± 7.50
Gender	
male	291 (51.1)
female	279 (48.9)
Residence	
rural	273 (47.9)
urban	297 (52.1)
Companion	
yes	436 (76.5)
no	134 (23.5)
Education	
primary school and below	513 (90.0)
middle school	34 (6.0)
High school or vocational school	20 (3.5)
College degree or above	3 (0.5)

### Selection of predictors

The 414 patients with sarcopenia from CHARLS (2011–2015) were used as the training and test sets for selecting predictors and model construction, focusing on respondents’ general information, health status, physical measurements, and cognitive status. Considering 30 drivers of MCI, all had 80% or more completeness of the data, and the expression levels of all variables were standardized. We use ML for feature double filtering. The random forest model was constructed using the R software random forest package to rank the importance of features and exclude those with mean decrease Gini (MDG) of less than 1. The remaining 10 features were subjected to recursive feature elimination (re) using the caret package, and in the process of elimination, ten-fold cross-validation was used to avoid overfitting. After the dual method validation, the final 8 features entered the next modeling stage, and these metrics included ASM, SMI, BMI, Grip, walk time, Depression, SPPB and sleep time (Table 2). Among them, muscle mass index played a crucial role in the model’s prediction, and ASM (*P* = 0.02) showed a significant difference between the MCI and non-MCI groups.

**Table 2:** Comparison of the characteristics of the population with and without MCI and ranking of the importance of the characteristics to the model

	Total(*n* = 414)	No(*n* = 329)	Yes(*n* = 85)	*P*-value	MDG	Variable importance(rfe)
ASM	13.58 ± 3.73	13.80 ± 5.78	12.75 ± 3.53	0.02	2.51	12.33
SMI	5.58 ± 1.01	5.62 ± 1.00	5.42 ± 1.01	0.10	1.82	12.29
BMI	18.58 ± 1.64	18.55 ± 1.67	18.68 ± 1.50	0.47	2.12	8.01
Grip	23.70 ± 8.14	23.86 ± 8.19	23.10 ± 7.95	0.44	1.64	6.19
Walk time	9.72 ± 4.03	9.80 ± 4.18	9.42 ± 3.37	0.38	2.66	6.09
Depression	9.75 ± 5.18	9.77 ± 5.15	9.66 ± 5.34	0.87	1.86	5.22
SPPB	7.86 ± 1.62	7.85 ± 1.58	7.91 ± 1.74	0.79	1.61	5.08
Sleep time	6.57 ± 2.26	6.61 ± 2.22	6.41 ± 2.43	0.50	1.70	3.94

### Model establishment

**Fig. 2:**
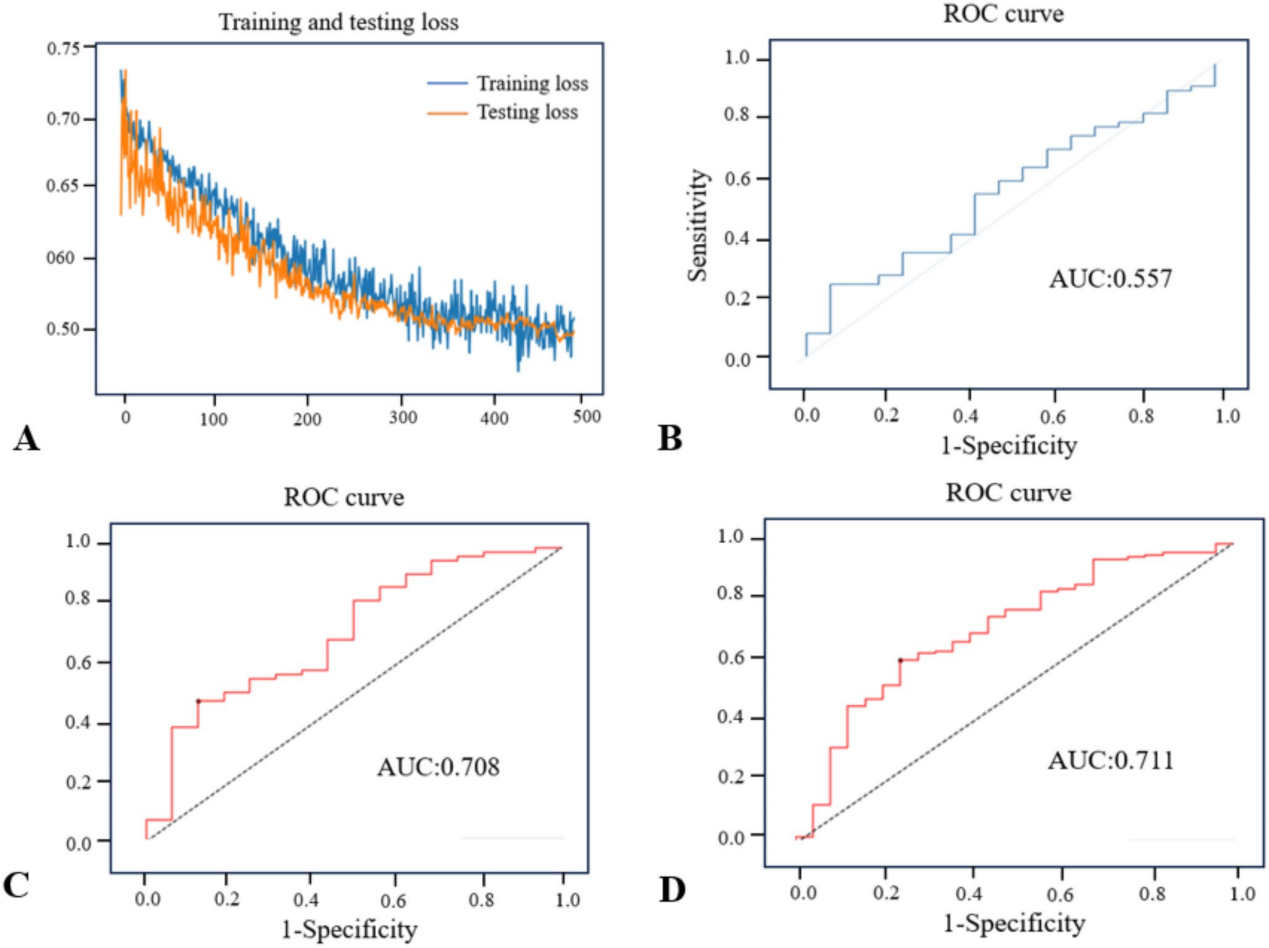
Loss curve and ROC curves of different data sets. **A** loss curve for the train and test set; **B** ROC curve of the DL model in test set; **C** ROC curve of logistic regression model in test set; **D** ROC curve of the DL model in validation

A 4-layer feed-forward neural network was constructed using DL, consisting of an input layer, 2 hidden layers, and an output layer, sigmoid as an activation function for neural nodes, constituting our MCI prediction model. 414 patients with sarcopenia were divided into a train set and a test set in a ratio of 8:2 for training and testing. The Loss curve shows that the DL model is well-fitted and does not appear to be under-trained or over-fitted. The loss curve for the train and test set is shown in Fig. 2. Due to the lack of control, we constructed a logistic regression model using the same variables.

### Performance of the prediction model

We use the AUC value of the area under the ROC curve to evaluate the model’s predictive performance. Our DL prediction model has an AUC of 0.708 (95% CI: 0.544–0.844) on the test set. The model’s accuracy was 0.542, precision was 0.939 and F1 was 0.633. Logistic regression has an AUC value 0.557 (0.95 CI, 0.410–0.705) on the test set. The ROC curves for the test set are shown in Fig. 2.

### Risk stratification

We further calculated the risk for each individual in the entire training cohort. The DL model gives an optimal risk threshold for predicting MCI or non-MCI. All patients were divided into two groups based on a 51.72% risk threshold. A total of 311 and 103 sarcopenia patients were classified into low-risk and high-risk groups, respectively, with the actual risk probability of critical illness events at 14.15% and 39.81%. There was a statistically significant difference between the low-risk and high-risk groups (*P* = 0.000). According to the DL model, the false-positive probability of onset in patients with sarcopenia at high risk of MCI in the next four years was only 10.23%, demonstrating the importance of the DL model in determining whether to intervene.

### Validation of the prediction model

Validation was performed using 156 patients with sarcopenia from the CHARLS cohort 2015–2018 who met inclusion criteria, of which 25 had a positive outcome. The AUC of the validation centralized model was 0.711 (0.95 CI, 0.588–0.823). The model’s accuracy was 0.628, precision was 0.929 and F1 was 0.737. The DL model showed relatively good performance in an independent validation set. The ROC curve for the validation set is shown in Fig. 2.

### Development of an online tool

**Fig. 3:**
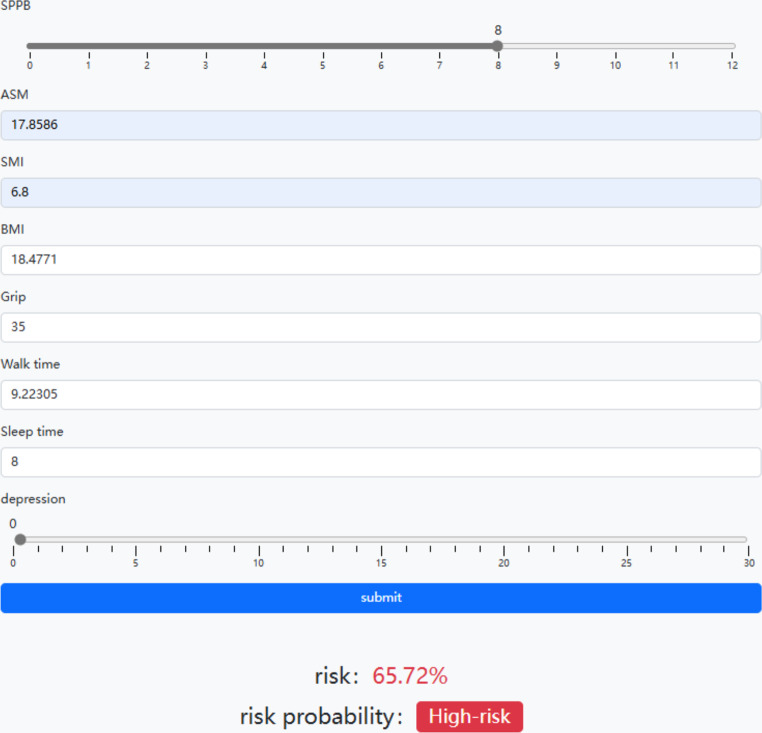
The application of on-line computing tool

To facilitate clinical application, we have developed an online calculation tool (http://47.115.214.16:8000/) for predicting the future risk of MCI in patients with sarcopenia. The tool calculates the risk based on physical measures and other relevant characteristics of patients. The online tool is shown in Fig. 3. The DL model demonstrates the feasibility and utility of predicting MCI in patients with sarcopenia.

All codes for feature selection, model construction, and other related processes is provided detailed in the supplementary material.

## Discussion

Sarcopenia and MCI are not independent conditions. They share numerous common pathogenic mechanisms and causative factors. A systematic evaluation of the prevalence of sarcopenia and MCI concluded that the overall prevalence of MCI in patients with sarcopenia was 20.5%, with high heterogeneity. In contrast, the overall prevalence of sarcopenia in patients with MCI was 9.1% [30], which suggests that the prevalence of MCI in patients with sarcopenia is relatively high and that sarcopenia may be a risk factor for MCI. Several longitudinal studies have shown a significant association between sarcopenia and MCI. The components of sarcopenia, especially grip strength and gait speed, can serve as predictors for early prediction and diagnosis of MCI. Therefore, we utilized the body measurements related to sarcopenia for risk prediction of MCI. The variables included were those associated with the onset of MCI, which confirmed in previous studies and easily measured daily. Since our model was designed with clinical consultation, we did not include more complex laboratory indicators related to MCI onset.

Our prediction of MCI in the specific population of sarcopenia, in addition to the high prevalence of MCI, is related to dementia, a severe adverse outcome caused by MCI. First, studies have shown that effective post-intervention can halve the risk of MCI in the next five years in patients with sarcopenia [11], which demonstrates the importance of screening people at high risk for MCI and providing early prevention. Second, 14.4-55.6% of patients with MCI may regain neurologic integrity [3], suggesting that early diagnosis of MCI is critical. Risk screening and early diagnosis and intervention may significantly reduce the incidence of dementia and reduce the medical and social burden. Cognitive impairment due to sarcopenia differs from other causes or primary cognitive impairment in that they have a particular physical burden that may not be amenable to interventions such as exercise to prevent or mitigate the progression of the disease. Therefore, we hope to use our model to accurately and promptly determine the risk of developing MCI. Further studies can set up personalized intervention programs for patients with sarcopenia who are at high risk of developing MCI and dementia to achieve precise intervention.

Regarding variable inclusion in the model, age and gender are considered variables strongly associated with the onset of MCI. The exclusion of age from the model may be due to the relatively narrow age of the sample. Factors such as ASM and SMI were also included in the model, as they are calculated from variables like age and gender. Although age, gender and similar factors were not directly not included in the DL model, their interactions with other variables play a critical role in shaping the model. Additionally, studies have shown that grip strength and gait speed are significant predictors of MCI, which is consistent with the characteristics selected for our models. Furthermore, studies indicates that the sleep duration and the presence of depressive symptoms are also associated with cognitive impairment. In patients with sarcopenia, both sleep time and depression may represent more important risk factors for accelerated MCI or dementia.

DL is a new research direction in ML that learns the intrinsic patterns and levels of representation of sample data. Neural networks discover distributed feature representations of data by combining low-level features to form more abstract high-level representations of attribute categories or features. Its essential feature is to try to mimic the pattern of transmitting and processing information between neurons in the brain by designing and establishing appropriate neuron computation nodes and multi-computing hierarchies, selecting proper input and output layers, and through learning and tuning of the network, establishing the input-to output functional relationship. The purpose of our choice of DL modeling is to take advantage of neural networks to learn and infer higher-order nonlinear associations between clinical features and patient outcomes in an entirely data-driven manner [31] and to deeply analyze the effects of variables and interactions between variables on outcomes, which is something that cannot be achieved by ordinary regression, and also, as shown in the results, compared with the basic regression, the feed-forward neural network does show better prediction results. In addition, with the continuous development of DL, its application in the medical industry is becoming more and more extensive. In the future, we can also use DL methods to develop disease-personalized interventions and care for patients.

The reason for the relatively significant difference in AUC values between the base regression model and the DL model may be the complexity of the neural network. The neural network can decrease the error along the gradient by the strength of the connection between the input node and the hidden node, the strength of the connection between the hidden node and the output node, and the threshold value. After repeated training and learning, the weight and threshold value corresponding to the minimum error can be determined. In addition, compared to single regression, neural networks can predict the effect of individual variables on the outcome and the effect of interactions between variables on the MCI.

Our strengths are that the DL model showed good performance in both model construction and validation, and our cohort samples are all over the country, which is also well generalized in China. Second, we used ML methods to double-validate the inclusion variables and performed model construction by combining DL and ML. Third, when searching for domestic and international studies, we could not find other studies that predicted MCI in patients with sarcopenia. Lastly, we built an online computational webpage to facilitate the application of the model.

The study has several limitations. First, we lacked external validation sets from foreign populations, which prevents us from confirming whether the model applies to other races and populations globally. We are actively seeking data from different countries and regions to address this issue as early as possible. Second, due to the limitations of public databases, variables related to the onset of MCI, such as the exercise situation, were not comprehensive enough due to the large missing amount and not being included in the study. Third, although BIA or DXA are generally used for measurement regarding the assessment of variables, the formula we used for ASM calculation aligns better with DXA. For walking speed, CHARLS uses measurements at a standard distance of five meters, whereas the international standard is a distance of six meters. However, it has been documented that this distance does not affect walking speed [32]. Finally, since the included samples were all individuals aged 45 and older, the DL model is mainly applicable to the elderly population and may not be relevant for predicting MCI in other age groups.

## Conclusion

We used CHARLS 2011–2015 data to construct the model, incorporating eight double-validated variables. Compared to logistic regression 0.557 (0.95 CI, 0.410–0.705), the DL model demonstrated strong predictive performance, with an AUC of 0.708 (95% CI: 0.544–0.844) for the test set. We further calculated the risk of morbidity for each individual in the training set and divided all patients into two groups based on 51.72% risk probability. A statistically significant difference in morbidity risk was observed across the low-risk and high-risk groups (*P* = 0.000). Additionally, the model showed a false-positive rate for MCI of 10.23% in high-risk sarcopenia populations, suggesting that these populations should receive immediate interventions to reduce the unnecessary burden. We used data from CHARLS 2015–2018 for independent validation, and the AUC value for the validation set was 0.711 (0.95 CI, 0.588–0.823). The DL model has demonstrated its feasibility and utility in predicting the risk of MCI in sarcopenia patients over the next 4 years (http://47.115.214.16:8000/). It provides clinical benefits in identifying sarcopenia patients at different MCI risk levels.

## Electronic supplementary material

Below is the link to the electronic supplementary material.

Supplementary Material 1

Supplementary Material 2

## Data Availability

No datasets were generated or analysed during the current study.
