# Supplementary material for: A prediction model for the risk of developing mild cognitive impairment in older adults with sarcopenia: evidence from the CHARLS
Source: Aging Clin Exp Res. 2025 Mar 8;37(1):69. doi: 10.1007/s40520-025-02980-2 (PMC11889009; doi:10.1007/s40520-025-02980-2)
Supplement: Supplementary file 1 — Supplementary Material 1 [file 40520_2025_2980_MOESM1_ESM.docx]

**A relatively accurate prediction model for the risk of developing mild cognitive impairment in patients with sarcopenia: Evidence from the CHARLS**

Xinyue Liu^a,b,#^, Jingyi Ni^a,#^, Baicheng Wang^a,#^, Rui Yin^a^, Jinlin Tang^a,b^, Qi Chu^c^, Lianghui You^a^, Zhenggang Wu^a^, Yan Cao^a^, Chenbo Ji^a,b,*^

^a^ Nanjing Women and Children's Healthcare Institute, Nanjing, Jiangsu, China.

^b^ School of Nursing, Nanjing Medical University, Nanjing, Jiangsu, China.

**^c^** Sunshine Union Hospital, Weifang, Shandong, China.

**^#^Co-first author**

**^*^Corresponding author:** chenboji@njmu.edu.cn (C.B. Ji).

**Journal**: Aging clinical and experimental research

**Data filtering**


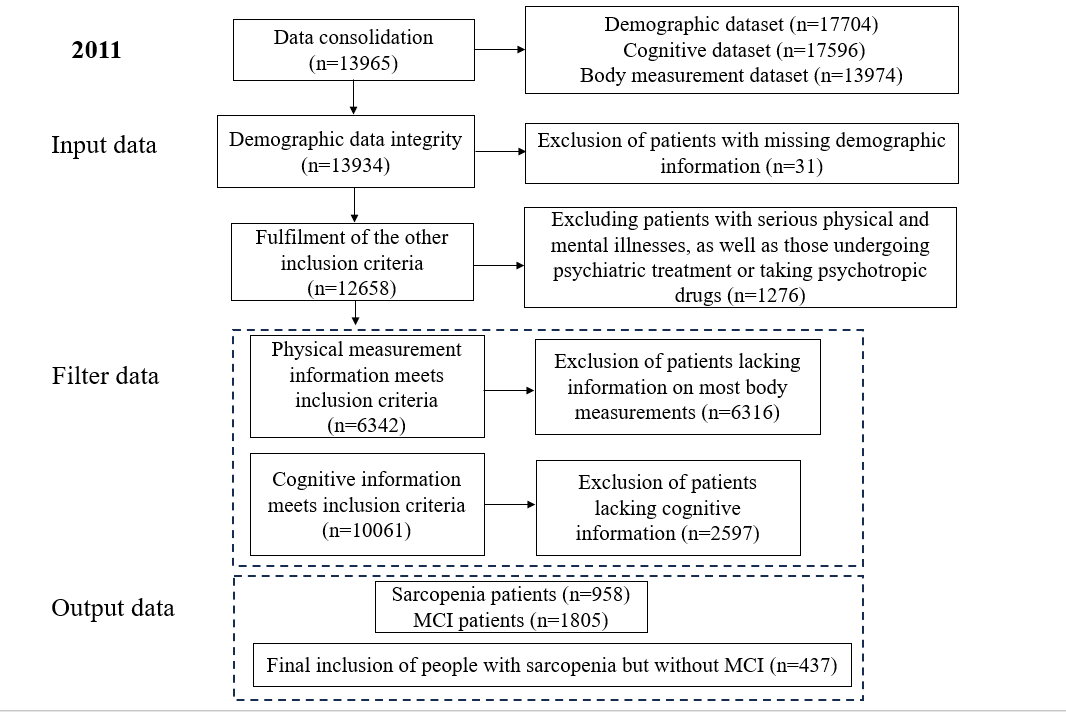


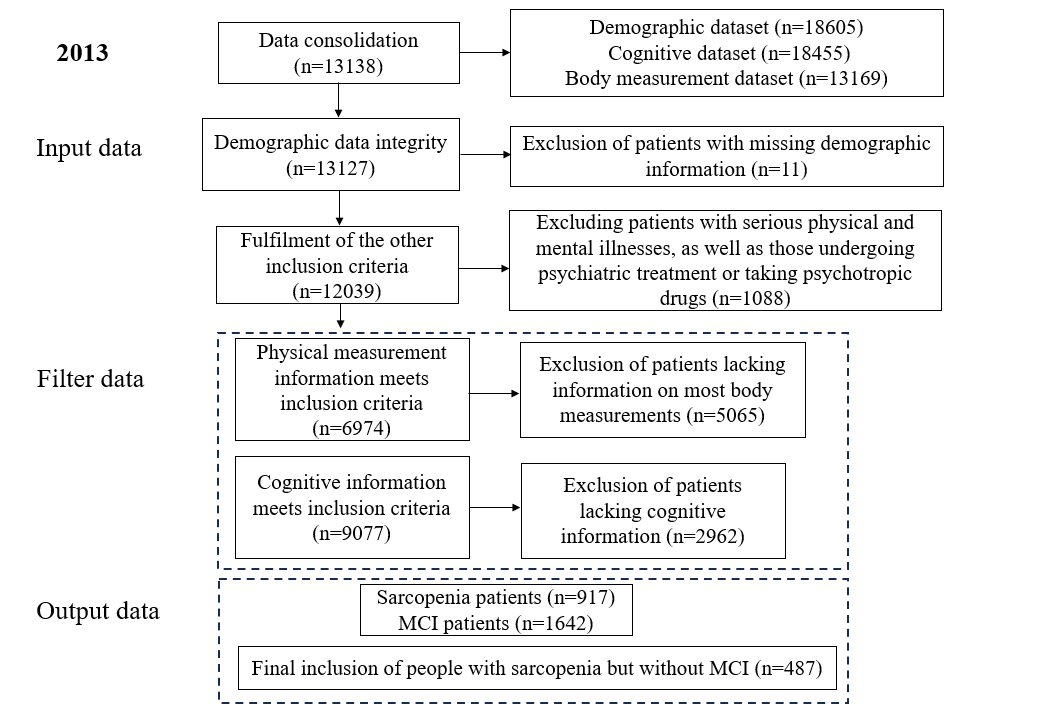


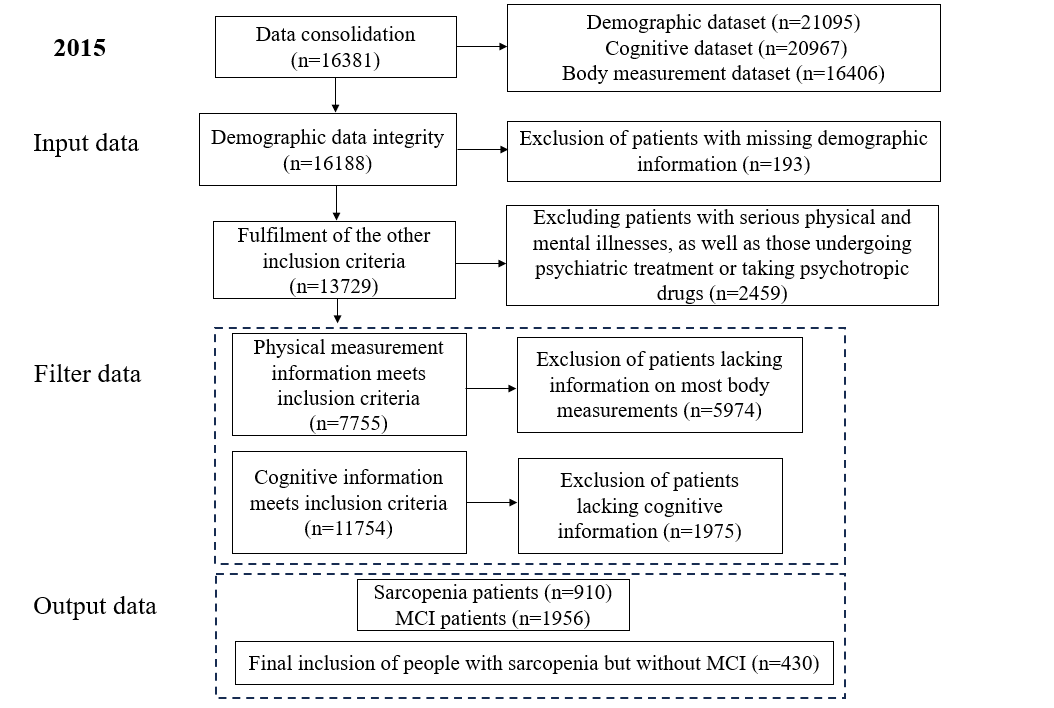


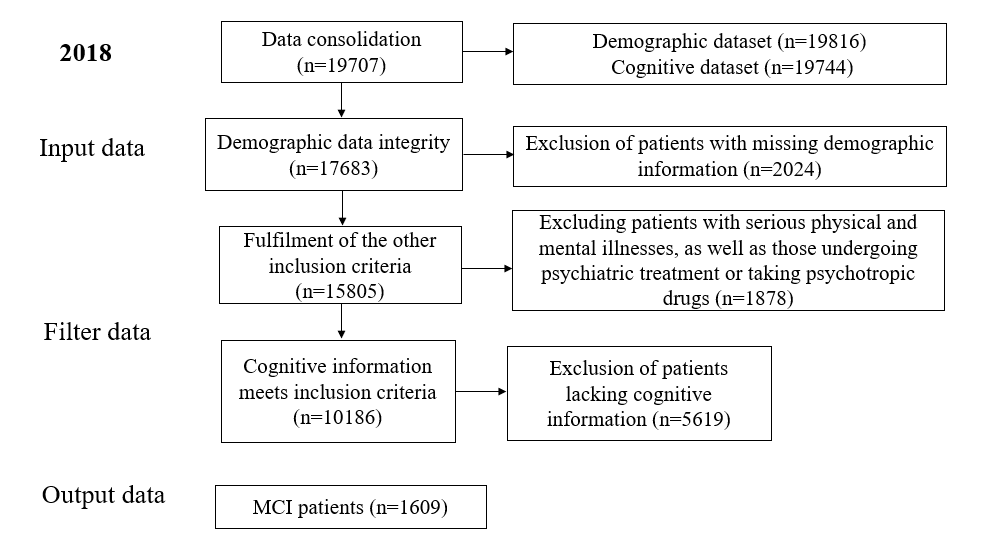


**Code**

**R padding (R 4.2.2)**

**library(missForest)**

**setwd("C:/data")**

**read.csv(file="data.csv",header = T)**

**P1<-read.csv(file="data.csv",header = T)**

**P1**

**sum(is.na(P1))**

**table(is.na(P1))**

**P1miss<-function(x){sum(is.na(x))/length(x)*100}**

**apply(P1,2,P1miss)**

**P1$residence<-factor(P1$residence)**

**dummy_edu1<-ifelse(P1$Education=="1",1,0)**

**dummy_edu2<-ifelse(P1$Education=="2",1,0)**

**dummy_edu3<-ifelse(P1$Education=="3",1,0)**

**P1$gender<-factor(P1$gender)**

**P1$marital<-factor(P1$marital)**

**P1$hypertension<-factor(P1$hypertension)**

**P1$dyslipidemia<-factor(P1$dyslipidemia)**

**P1$diabetes<-factor(P1$diabetes)**

**P1$ malignancy<-factor(P1$malignancy)**

**P1$ chroniclungdisease<-factor(P1$ chroniclungdisease)**

**P1$liverdisease<-factor(P1$liverdisease)**

**P1$heartdisease<-factor(P1$heartdisease)**

**P1$stroke<-factor(P1$stroke)**

**P1$kidneydisease<-factor(P1$kidneydisease)**

**P1$stomachdisease<-factor(P1$stomachdisease)**

**P1$arthritis<-factor(P1$arthritis)**

**P1$asthma<-factor(P1$asthma)**

**P1$fall<-factor(P1$fall)**

**P1$smoke<-factor(P1$smoke)**

**P1$drink<-factor(P1$drink)**

**summary(P1)**

**P1.imp<-missForest(P1)**

**P1.imp**

**sum(is.na(P1.imp))**

**P1.imp$ximp**

**P1.imp$OOBerror**

**class(P1.imp$ximp)**

**write.csv(P1.imp$ximp,file="C:/data/2011.csv",quote=F,row.names=F)**

**write.csv(P1.imp$ximp,file="C:/data/2013.csv",quote=F,row.names=F)**

**write.csv(P1.imp$ximp,file="C:/data/2015.csv",quote=F,row.names=F)**

**write.csv(P1.imp$ximp,file="C:/data/2018.csv",quote=F,row.names=F)**

**Random forest: feature selection**

**library(rio)**

**library(ggplot2)**

**library(magrittr)**

**library(randomForest)**

**library(tidyverse)**

**library(skimr)**

**library(caret)**

**library(varSelRF)**

**library(pdp)**

**library(iml)**

**library(readxl)**

**B1<-read_excel("D:/R-4.2.2/library/randomForest/train.xlsx",sheet = "Sheet1")**

**as.data.frame(B1)**

**B1$gender<-factor(B1$gender,labels=c("male","female"))**

**B1$residence<-factor(B1$residence,labels=c("rural","urban"))**

**B1$marital<-factor(B1$marital,labels=c("yes","no"))**

**B1$hypertension<-factor(B1$hypertension,labels=c("yes","no"))**

**B1$dyslipidemia<-factor(B1$dyslipidemia,labels=c("yes","no"))**

**B1$diabetes<-factor(B1$diabetes,labels=c("yes","no"))**

**B1$malignancy<-factor(B1$malignancy,labels=c("yes","no"))**

**B1$chroniclungdisease<-factor(B1$chroniclungdisease,labels=c("yes","no"))**

**B1$liverdisease<-factor(B1$liverdisease,labels=c("yes","no"))**

**B1$heartdisease<-factor(B1$heartdisease,labels=c("yes","no"))**

**B1$stroke<-factor(B1$stroke,labels=c("yes","no"))**

**B1$kidneydisease<-factor(B1$kidneydisease,labels=c("yes","no"))**

**B1$stomachdisease<-factor(B1$stomachdisease,labels=c("yes","no"))**

**B1$arthritis<-factor(B1$arthritis,labels=c("yes","no"))**

**B1$asthma<-factor(B1$asthma,labels=c("yes","no"))**

**B1$smoke<-factor(B1$smoke,labels=c("yes","no"))**

**B1$drink<-factor(B1$drink,labels=c("yes","no"))**

**B1$fall<-factor(B1$fall,labels = c("yes","no"))**

**B1$mci<-factor(B1$mci, labels=c("yes","no"))**

**dummy_edu1<-ifelse(B1$education=="1",1,0)**

**dummy_edu2<-ifelse(B1$education=="2",1,0)**

**dummy_edu3<-ifelse(B1$education=="3",1,0)**

**trainsub = sample(nrow(B1),8/10*nrow(B1))**

**traindata = B1[trainsub,]**

**testdata = B1[-trainsub,]**

**form_reg<-as.formula(paste0("mci~",**

**paste(colnames(traindata)[1:30],collapse = "+")))**

**form_reg**

**set.seed(1234)**

**n<-ncol(B1)-5**

**errRate<-c(1)**

**for(i in 1:n){**

**rf_train<-randomForest(form_reg,data=traindata,**

**ntree=1000,**

**p=0.7,**

**mtry=i,**

**importance=T)**

**errRate[i]<-mean(rf_train$err.rate)**

**print(rf_train)**

**}**

**m=which.min(errRate)**

**print(m)**

**set.seed(1234)**

**rf_train<-randomForest(form_reg,data=traindata,**

**mtry=1,ntree=500,importance=T,proximity=TRUE)**

**rf_train**

**plot(rf_train,main="ERROR & TREES")**

**rf<-randomForest(form_reg,data = traindata,ntree =100,mtry=1,importance=TRUE)**

**rf**

**importance(rf)**

**varImpPlot(rf,main = "variable importance", n.var = 30)**

**
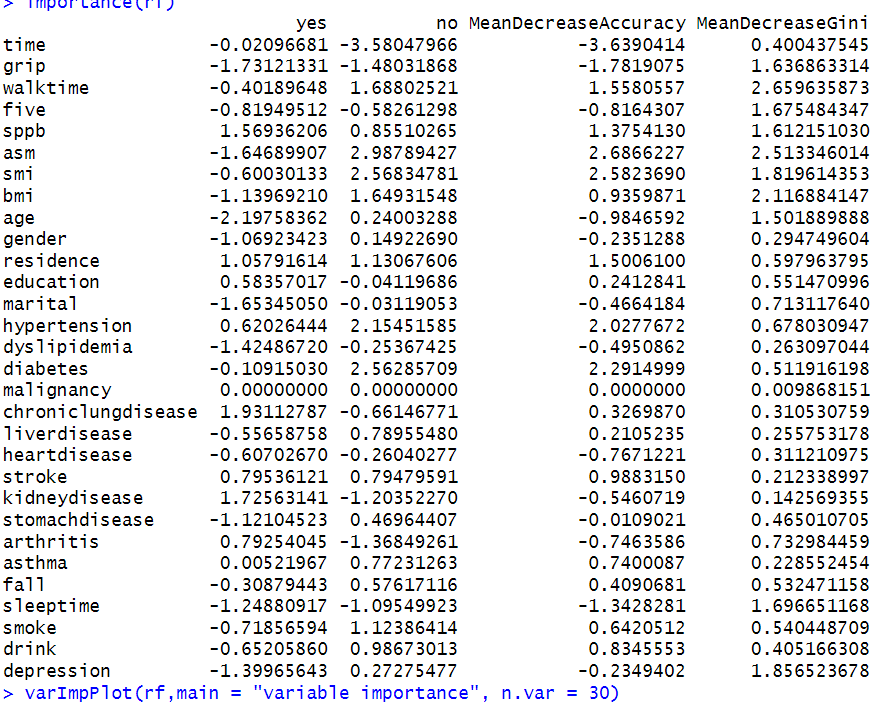
** **
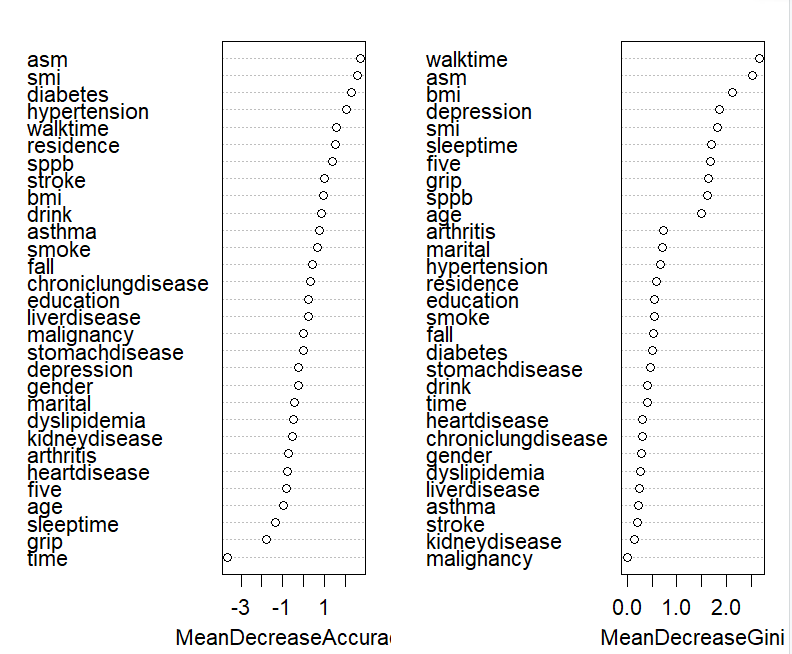
**

**Feature elimination**

**library(readxl)**

**P1<-read_excel("D:/R-4.2.2/library/caret/data/train.xlsx",sheet = "Sheet1")**

**P2<-c(1,1,1,2,1,2,1,2,2,1,1,2,1,2,1,2,2,1,1,2,2,2,2,2,1,2,2,2,1,2,2,2,2,2,1,2,2,1,2,1,1,2,2,2,2,1,2,2,2,2,2,2,2,2,1,2,2,2,2,2,1,1,2,2,2,2,2,2,2,1,2,2,2,2,2,2,2,2,2,2,2,1,1,2,1,2,2,2,1,2,2,1,2,2,2,1,2,2,1,2,2,2,2,2,2,2,2,2,2,1,2,2,2,2,2,2,2,1,2,2,2,2,2,2,2,2,1,2,2,2,2,2,2,2,2,2,2,2,2,1,1,1,1,2,2,1,2,2,2,2,2,2,2,2,2,1,2,2,2,2,2,1,2,2,2,1,1,2,2,2,1,2,2,1,2,2,2,2,1,2,2,2,2,2,1,2,2,1,2,2,1,2,2,2,2,2,2,2,2,2,2,2,1,2,2,2,1,2,2,2,2,2,2,2,2,1,2,2,2,2,2,2,2,2,2,2,2,2,2,1,2,2,2,2,2,2,2,2,1,2,2,1,2,2,2,2,1,2,2,2,2,2,2,2,2,2,2,2,2,2,2,2,2,1,2,1,1,2,2,1,1,2,2,2,2,2,1,2,2,2,2,2,1,2,2,2,2,2,2,2,2,2,2,1,2,2,2,2,2,2,2,2,2,1,2,2,2,2,2,2,2,2,2,2,2,2,2,2,2,2,2,2,1,2,2,2,1,2,2,2,1,1,2,2,2,1,1,2,2,2,2,2,2,2,2,1,2,2,2,2,1,1,2,2,1,2,2,2,2,2,2,2,2,2,1,2,2,2,2,1,2,2,2,2,2,2,2,2,2,2,2,1,2,1,2,1,2,2,1,1,2,1,2,2,2,2,2,2,2,2,2,2,2,2,2,2,2,1,1,2,1,1,2,2)**

**library(caret)**

**set.seed(1234)**

**rfeControl = rfeControl(functions = rfFuncs,**

**method = "cv",**

**number = 10**

**)**

**library(future)**

**plan("multisession",workers=8)**

**rfProfile <- rfe(P1, P2,**

**sizes = c(1:10),**

**rfeControl = rfeControl,**

**allowParallel = T**

**)**

**varImp(rfProfile)**

**
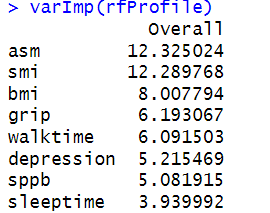
**

**Logistic regression model**

**library(rms)**

**library(mice)**

**library(autoReg)**

**library(caret)**

**library(pROC)**

**library(rmda)**

**library(dplyr)**

**library(rrtable)**

**library(foreign)**

**library(regplot)**

**library(readxl)**

**A1<-read_excel("D:/R-4.2.2/library/rms/train.xlsx",sheet = "Sheet1")**

**x <- scale(A1[,-nearZeroVar(A1)])**

**x <- x[, -findCorrelation(cor(x), .8)]**

**x <- as.data.frame(x, stringsAsFactors = TRUE)**

**A1<-as.data.frame(A1)**

**head(A1)**

**str(A1)**

**A1$residence<-factor(A1$residence,labels=c("yes","no"))**

**A1$diabetes<-factor(A1$diabetes,labels=c("yes","no"))**

**A1$mci<-factor(A1$mci, labels=c("yes","no"))**

**head(A1)**

**str(A1)**

**attach(A1)**

**training.sample<-A1$mci %>% createDataPartition(p=0.8,list=FALSE)**

**train.data<-A1[training.sample,]**

**test.data<-A1[-training.sample,]**

**dd <- datadist(train.data)**

**options(datadist="dd")**

**fit1<-lrm(mci~.,data=train.data,x=T,y=T)**

**fit1**

**train_pred<-predict(fit1,newdata=train.data,type="fitted")**

**test_pred<-predict(fit1,newdata=test.data,type="fitted")**

**test_roc<-roc(test.data$mci,test_pred)**

**auc(test_roc)**

**ci(test_roc)**

**plot(test_roc)**

**
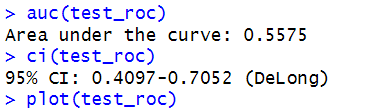
**

**
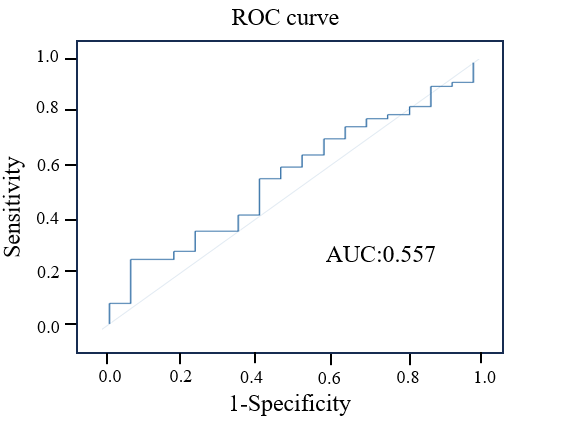
**

**Neural network: DL mode (Pytorch2.2.1)**

**import torch**

**from torch import nn,optim**

**import torch.utils.data as Data**

**import numpy as np**

**import pandas as pd**

**import os ,re**

**import random**

**import copy**

**import matplotlib.pylab as plt**

**plt.rcParams['font.sans-serif']=['SimHei']**

**plt.rcParams['axes.unicode_minus']=False**

**import sklearn.preprocessing as preprocessing**

**from sklearn.model_selection import train_test_split**

**from sklearn.metrics import f1_score,recall_score,precision_score,roc_curve,roc_auc_score,accuracy_score,confusion_matrix**

**from sklearn.preprocessing import StandardScaler**

**import gc**

**import joblib**

**p = 0.7**

**l = 0.0005**

**Epochs = 500**

**input_size = 8**

**output_size = 1**

**df = pd.read_excel('train.xlsx')**

**df['mci'] = df['mci'].apply(lambda x:0 if x==1 else 1)**

**df=df.iloc[:,1:]**

**y = df['mci']**

**df = df.drop(labels = 'mci',axis = 1)**

**df['mci'] = y**

**df = df.dropna()**

**df.shape**

**df = df.astype('float')**

**df**

**X_train,X_test, Y_train, Y_test =train_test_split(df.iloc[:,:-1],df.iloc[:,-1],test_size=0.2, random_state=0)**

**def z_score(data):**

**data = data.astype(float)**

**Mean = data.mean()**

**Var = ((data - Mean)**2).mean()**

**Std = pow(Var,0.5)**

**data = (data - Mean)/Std**

**return Mean,Std,data**

**Mean,Std,_ = z_score(X_train.iloc[:,:6])**

**import joblib**

**joblib.dump(Mean, 'Mean')**

**joblib.dump(Std, 'Std')**

**Mean**

**df.iloc[:,:6] = (df.iloc[:,:6] - Mean)/Std**

**df**

**Y_test**

**X_train,X_test, Y_train, Y_test =train_test_split(df.iloc[:,:-1].values,df.iloc[:,-1].values,test_size=0.2, random_state=6)**

**from imblearn.over_sampling import RandomOverSampler**

**over_sampler= RandomOverSampler()**

**X_train,Y_train = over_sampler.fit_resample(X_train,Y_train)**

**print(X_train.shape, Y_train.shape,X_test.shape, Y_test.shape)**

**X_train = torch.tensor(X_train,dtype = torch.float)**

**Y_train = torch.tensor(Y_train,dtype = torch.float)**

**X_test = torch.tensor(X_test,dtype = torch.float)**

**Y_test = torch.tensor(Y_test,dtype = torch.float)**

**train_loader = Data.DataLoader(**

**dataset=Data.TensorDataset(X_train, Y_train),**

**batch_size=10,**

**shuffle=True,**

**drop_last =True,**

**num_workers=0,**

**)**

**test_loader = Data.DataLoader(**

**dataset=Data.TensorDataset(X_test, Y_test),**

**batch_size=32,**

**shuffle=False,**

**drop_last =True,**

**num_workers=0,**

**)**

**def seed_torch():**

**seed=1029**

**random.seed(seed)**

**os.environ['PYTHONHASHSEED'] = str(seed)**

**np.random.seed(seed)**

**torch.manual_seed(seed)**

**torch.cuda.manual_seed(seed)**

**torch.cuda.manual_seed_all(seed)**

**class BP(nn.Module):**

**def __init__(self,p,input_size, output_size):**

**super().__init__()**

**self.linear1 = nn.Linear(input_size, 128)**

**self.linear2 = nn.Linear(128, 64)**

**self.linear3 = nn.Linear(64, output_size)**

**self.dropout = nn.Dropout(p=p)**

**self.relu = nn.ReLU()**

**def forward(self, x):**

**x = self.relu(self.linear1(x))**

**x = self.dropout(x)**

**x = self.relu(self.linear2(x))**

**x = self.linear3(x)**

**x = torch.sigmoid(x)**

**x = torch.squeeze(x)**

**return x**

**device = torch.device("cuda:0" if torch.cuda.is_available() else "cpu")**

**seed_torch()**

**model = BP(p,input_size,output_size)**

**model = model.to(device)**

**loss_function = nn.BCELoss()**

**optimizer = torch.optim.Adam(model.parameters(), lr=l)**

**scheduler = optim.lr_scheduler.StepLR(optimizer, step_size=9, gamma=0.95)**

**loss_dict = {}**

**loss_dict['train_loss'] = []**

**loss_dict['test_loss'] = []**

**loss_dict['train_acc'] = []**

**loss_dict['test_acc'] = []**

**loss_dict['train_auc'] = []**

**loss_dict['test_auc'] = []**

**AUC= 0**

**LOSS_TEST = 100**

**Len_train = len(train_loader)**

**Len_test = len(test_loader)**

**for epochs in range(Epochs):**

**loss_mean_train = 0**

**loss_mean_test = 0**

**acc_train = 0**

**acc_test = 0**

**Brier_train = 0**

**Brier_test = 0**

**auc_train = 0**

**auc_test = 0**

**model.train()**

**for data_l in train_loader:**

**seq, labels = data_l**

**seq, labels = seq.to(device), labels.to(device)**

**optimizer.zero_grad()**

**y_pred = model(seq)**

**single_loss = loss_function(y_pred, labels)**

**single_loss.backward()**

**optimizer.step()**

**loss_mean_train += single_loss.item()**

**acc = accuracy_score(labels.cpu().numpy(), torch.round(y_pred).cpu().detach().numpy() )**

**acc_train += acc**

**try:**

**Auc = roc_auc_score(labels.cpu().numpy(), y_pred.cpu().detach().numpy())**

**except:**

**Auc = 0**

**auc_train +=Auc**

**scheduler.step()**

**loss_mean_train = loss_mean_train/Len_train**

**acc_train= acc_train/Len_train**

**auc_train = auc_train/Len_train**

**loss_dict['train_loss'].append(loss_mean_train)**

**loss_dict['train_acc'].append(acc_train)**

**loss_dict['train_auc'].append(auc_train)**

**model.eval()**

**y_pred = model(X_test)**

**loss_mean_test = loss_function(y_pred, Y_test).item()**

**acc_test = accuracy_score(Y_test.cpu().numpy(), torch.round(y_pred).cpu().detach().numpy())**

**try:**

**auc_test = roc_auc_score(Y_test.cpu().numpy(), y_pred.cpu().detach().numpy())**

**except:**

**auc_test = 0**

**loss_dict['test_loss'].append(loss_mean_test)**

**loss_dict['test_acc'].append(acc_test)**

**loss_dict['test_auc'].append(auc_test)**

**print('Epochs',epochs,'loss_train',round(loss_mean_train,5),'loss_test',round(loss_mean_test,5),)**

**print('acc_train',round(acc_train,3),'acc_test',round(acc_test,3))**

**print('auc_train',round(auc_train,3),'auc_test',round(auc_test,3))**

**print('------------------')**

**if AUC < auc_test:**

**AUC =auc_test**

**torch.save(model, 'model_bp1.pth')**

**print('已更新保存模型')**

**del seq, labels,y_pred**

**gc.collect()**

**torch.cuda.empty_cache()**

**import matplotlib.pyplot as plt**

**plt.figure(dpi=150,figsize=(7,4))**

**plt.plot(loss_dict['train_loss'][:], label='train')**

**plt.plot(loss_dict['test_loss'][:], label='test')**

**plt.xlabel('iteration')**

**plt.ylabel('logloss')**

**plt.title('bp_Training and Testing Loss')**

**plt.legend()**

**plt.savefig('lstm_loss.jpg',dpi=150)**

**plt.show()**

**
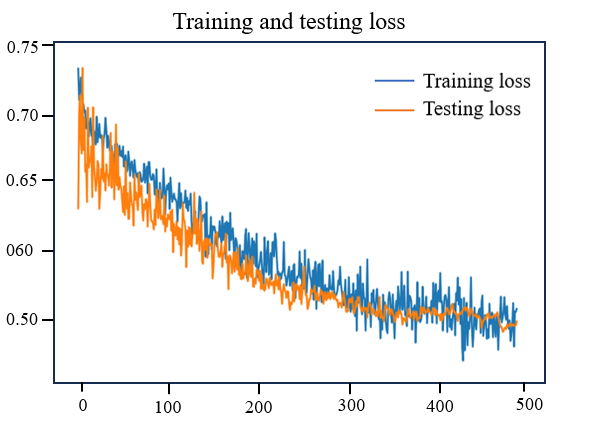
**

**device = torch.device("cuda" if torch.cuda.is_available() else "cpu")**

**model = torch.load('model_bp1.pth')**

**model = model.to(device)**

**yhat = model(X_test).cpu().detach().numpy()**

**yhat**

**Y = Y_test.cpu().detach().numpy().copy()**

**torch.cuda.empty_cache()**

**FPR,recall,thresholds = roc_curve(Y,yhat,pos_label=1)**

**area =roc_auc_score(Y,yhat)**

**maxindex = (recall-FPR).tolist().index(max(recall-FPR))**

**recall[maxindex]**

**prob = pd.DataFrame(yhat)**

**prob.loc[prob.iloc[:,0]>=thresholds[maxindex],'y_pred'] = 1**

**prob.loc[prob.iloc[:,0]<thresholds[maxindex],'y_pred'] = 0**

**print(thresholds[maxindex])**

**plt.figure(figsize=(10,7))**

**plt.plot(FPR,recall,color='red',**

**label='ROC curve(area=%0.3f)'%area)**

**plt.plot([0,1],[0,1],color='black',linestyle='--')**

**plt.scatter(FPR[maxindex],recall[maxindex],c='black',s=20)**

**r = '%0.3f' % recall[maxindex]**

**plt.text(FPR[maxindex]-0.05, recall[maxindex]+0.02, r,ha='center', va='bottom', fontsize=10)**

**plt.xlabel('False Positive Rate',fontsize = 15)**

**plt.ylabel('Recall',fontsize = 15)**

**plt.legend(loc='lower right')**

**plt.savefig(fname="ROC.jpg",dpi=500)**

**plt.show()**

**Predict = [1 if yhat[j]>thresholds[maxindex] else 0 for j in range(len(Y)) ]**

**from sklearn.metrics import confusion_matrix as CM, precision_score as P, recall_score as R**

**cm = CM(Y,Predict,labels=[1,0])**

**ACC= (cm[0,0]+cm[1,1])/cm[:,:].sum()**

**precision = cm[0,0]/(cm[0,0]+cm[1,0])**

**F1 = 2/(1/precision+1/recall[maxindex])**

**dict_h = {**

**'accuracy':ACC,**

**'precision':precision,**

**'recall':recall[maxindex],**

**'F1':F1,**

**'area':area,**

**}**

**df_h = pd.DataFrame(dict_h,index =[0])**

**df_h.to_excel('Assessment indicators.xlsx')**

**df_h**

**from sklearn.metrics import roc_auc_score**

**from sklearn.utils import resample**

**import numpy as np**

**def bootstrap_auc(y_true, y_scores, n_bootstraps=1000):**

**bootstrapped_scores = []**

**rng = np.random.RandomState(42) # Seed for reproducibility**

**for _ in range(n_bootstraps):**

**indices = rng.randint(0, len(y_scores), len(y_scores))**

**if len(np.unique(y_true[indices])) < 2:**

**continue**

**score = roc_auc_score(y_true[indices], y_scores[indices])**

**bootstrapped_scores.append(score)**

**sorted_scores = np.sort(bootstrapped_scores)**

**lower = np.percentile(sorted_scores, 2.5)**

**upper = np.percentile(sorted_scores, 97.5)**

**return lower, upper**

**lower, upper = bootstrap_auc(Y, yhat)**

**print(' AUC95% CI',lower, upper)**

**print(dict_h)**

**
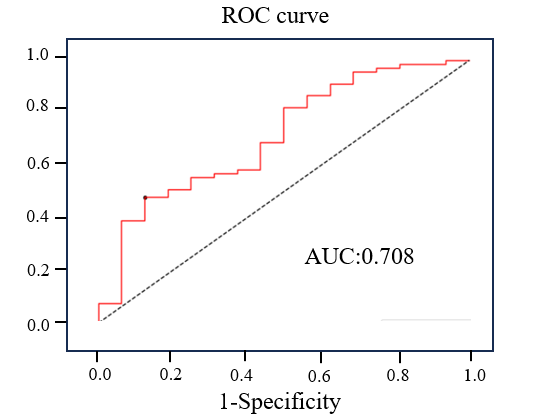
**

**dff = pd.read_excel('validation.xlsx')**

**dff['mci'] = dff['mci'].apply(lambda x:0 if x==1 else 1)**

**dff=dff.iloc[:,1:]**

**df**

**y = dff['mci']**

**dff = dff.drop(labels = 'mci',axis = 1)**

**dff['mci'] = y**

**dff.iloc[:,:6] = (dff.iloc[:,:6] - Mean)/Std**

**class BP(nn.Module):**

**def __init__(self,p,input_size, output_size):**

**super().__init__()**

**self.linear1 = nn.Linear(input_size, 256)**

**self.linear2 = nn.Linear(256, 64)**

**self.linear3 = nn.Linear(64, output_size)**

**self.dropout = nn.Dropout(p=p)**

**self.relu = nn.ReLU()**

**def forward(self, x):**

**x = self.relu(self.linear1(x))**

**x = self.dropout(x)**

**x = self.relu(self.linear2(x))**

**x = self.linear3(x)**

**x = torch.sigmoid(x)**

**x = torch.squeeze(x)**

**return x**

**dff = dff.astype('float')**

**x = dff.iloc[:,:-1].values**

**y = dff.iloc[:,-1].values**

**x = torch.tensor(x,dtype = torch.float)**

**device = torch.device("cuda" if torch.cuda.is_available() else "cpu")**

**model = torch.load('model_bp1.pth')**

**model = model.to(device)**

**yhat = model(x).cpu().detach().numpy()**

**Y = y**

**dff = pd.DataFrame()**

**dff['预测结果'] = yhat**

**dff['真实值'] = Y**

**dff.to_excel('验证集预测结果.xlsx',index =False)**

**torch.cuda.empty_cache()**

**FPR,recall,thresholds = roc_curve(Y,yhat,pos_label=1)**

**area =roc_auc_score(Y,yhat)**

**maxindex = (recall-FPR).tolist().index(max(recall-FPR))**

**recall[maxindex]**

**prob = pd.DataFrame(yhat)**

**prob.loc[prob.iloc[:,0]>=thresholds[maxindex],'y_pred'] = 1**

**prob.loc[prob.iloc[:,0]<thresholds[maxindex],'y_pred'] = 0**

**plt.figure(figsize=(10,7))**

**plt.plot(FPR,recall,color='red',**

**label='ROC curve(area=%0.3f)'%area)**

**plt.plot([0,1],[0,1],color='black',linestyle='--')**

**plt.scatter(FPR[maxindex],recall[maxindex],c='black',s=20)**

**r = '%0.3f' % recall[maxindex]**

**plt.text(FPR[maxindex]-0.05, recall[maxindex]+0.02, r,ha='center', va='bottom', fontsize=10)**

**plt.xlabel('False Positive Rate',fontsize = 15)**

**plt.ylabel('Recall',fontsize = 15)**

**# plt.title('Receiver operating characteristic example',fontsize = 20)**

**plt.legend(loc='lower right')**

**plt.savefig(fname="ROC.jpg",dpi=500)**

**plt.show()**

**Predict = [1 if yhat[j]>thresholds[maxindex] else 0 for j in range(len(Y)) ]**

**from sklearn.metrics import confusion_matrix as CM, precision_score as P, recall_score as R**

**cm = CM(Y,Predict,labels=[1,0])**

**ACC= (cm[0,0]+cm[1,1])/cm[:,:].sum()**

**precision = cm[0,0]/(cm[0,0]+cm[1,0])**

**F1 = 2/(1/precision+1/recall[maxindex])**

**dict_h = {**

**'准确率':ACC,**

**'精确度':precision,**

**'召回率':recall[maxindex],**

**'F1':F1,**

**'area':area,**

**}**

**# print(dict_h)**

**df_h = pd.DataFrame(dict_h,index =[0])**

**df_h.to_excel('验证集评估指标.xlsx')**

**df_h**

**from sklearn.metrics import roc_auc_score**

**from sklearn.utils import resample**

**import numpy as np**

**def bootstrap_auc(y_true, y_scores, n_bootstraps=1000):**

**bootstrapped_scores = []**

**rng = np.random.RandomState(42) # Seed for reproducibility**

**for _ in range(n_bootstraps):**

**indices = rng.randint(0, len(y_scores), len(y_scores))**

**if len(np.unique(y_true[indices])) < 2:**

**continue**

**score = roc_auc_score(y_true[indices], y_scores[indices])**

**bootstrapped_scores.append(score)**

**sorted_scores = np.sort(bootstrapped_scores)**

**lower = np.percentile(sorted_scores, 2.5)**

**upper = np.percentile(sorted_scores, 97.5)**

**return lower, upper**

**lower, upper = bootstrap_auc(Y, yhat)**

**print('验证集AUC95%可信区间',lower, upper)**

**print(dict_h)**

**
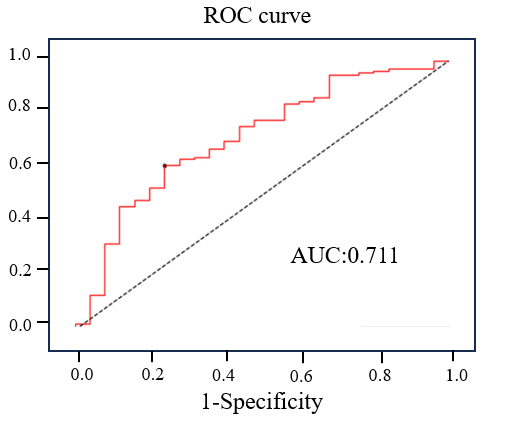
**

**dff = pd.read_excel('train.xlsx')**

**dff['mci'] = dff['mci'].apply(lambda x:0 if x==1 else 1)**

**dff=dff.iloc[:,1:]**

**dff**

**y = dff['mci']**

**dff = dff.drop(labels = 'mci',axis = 1)**

**dff['mci'] = y**

**dff.iloc[:,:6] = (dff.iloc[:,:6] - Mean)/Std**

**class BP(nn.Module):**

**def __init__(self,p,input_size, output_size):**

**super().__init__()**

**self.linear1 = nn.Linear(input_size, 256)**

**self.linear2 = nn.Linear(256, 64)**

**self.linear3 = nn.Linear(64, output_size)**

**self.dropout = nn.Dropout(p=p)**

**self.relu = nn.ReLU()**

**def forward(self, x):**

**x = self.relu(self.linear1(x))**

**x = self.dropout(x)**

**x = self.relu(self.linear2(x))**

**x = self.linear3(x)**

**x = torch.sigmoid(x)**

**x = torch.squeeze(x)**

**return x**

**dff = dff.astype('float')**

**x = dff.iloc[:,:-1].values**

**y = dff.iloc[:,-1].values**

**x = torch.tensor(x,dtype = torch.float)**

**device = torch.device("cuda" if torch.cuda.is_available() else "cpu")**

**model = torch.load('model_bp1.pth')**

**model = model.to(device)**

**yhat = model(x).cpu().detach().numpy()**

**yhat**
